# The impact of primary tumor sidedness on survival in early‐onset colorectal cancer by stage: A National Veterans Affairs retrospective analysis

**DOI:** 10.1002/cam4.3757

**Published:** 2021-04-02

**Authors:** Ibrahim Azar, Nada Al Masalmeh, Saghi Esfandiarifard, Gurjiwan Virk, Wissam Kiwan, Anthony Frank Shields, Syed Mehdi, Philip A. Philip

**Affiliations:** ^1^ Karmanos Cancer Institute Detroit MI USA; ^2^ Wayne State University Detroit MI USA; ^3^ Albany Medical College Albany NY USA; ^4^ University of Maryland Baltimore MD USA; ^5^ Stratton Veterans’ Affairs Medical Center Albany NY USA

**Keywords:** colon cancer, colorectal cancer, early‐onset colorectal cancer, laterality, left‐sided colon cancer, primary tumor sidedness, right‐sided colon cancer

## Abstract

**Background:**

The incidence of early‐onset colorectal cancer (EOCRC) is rising. Left‐sided colorectal cancer (LCC) is associated with better survival compared to right‐sided colon cancer (RCC) in metastatic disease. NCCN guidelines recommend the addition of EGFR inhibitors to KRAS/NRAS WT metastatic CRC originating from the left only. Whether laterality impacts survival in locoregional disease and EOCRC is of interest.

**Methods:**

65,940 CRC cases from the National VA Cancer Cube Registry (2001–2015) were studied. EOCRC (2096 cases) was defined as CRC diagnosed at <50 years. Using ICD codes, RCC was defined from the cecum to the hepatic flexure (C18.0–C18.3), and LCC from the splenic flexure to the rectum (C18.5–18.7; C19 and C20).

**Results:**

EOCRC is more likely to originate from the left side (66.65% LCC in EOCRC vs. 58.77% in CRC). Overall, LCC has better 5‐year Overall Survival (OS) than RCC in stages I (61.67% vs. 58.01%) and III (46.1% vs. 42.1%) and better 1‐year OS in stage IV (57.79% vs. 49.49%). Stage II RCC has better 5‐year OS than LCC (53.39% vs. 49.28%). In EOCRC, there is no statistically significant difference between LCC and RCC in stages I‐III. Stage IV EOCRC patients with LCC and RCC have a 1‐year OS of 73.23% and 59.84%, respectively.

**Conclusion:**

In EOCRC, LCC is associated with better OS than RCC only stage IV. In the overall population, LCC is associated with better OS in all stages except stage II. The better prognosis of stage II RCC might be due to the high incidence of mismatch repair deficient tumors in this subpopulation.

## Graphical abstract

In EOCRC patients, LCC is associated with better OS than RCC only in patients with metastatic disease. In the overall population, LCC is associated with better OS in all stages except stage II, which might be due to the high incidence of MMRd tumors in this subpopulation.

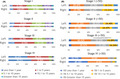

**Lay Summary**

Colorectal cancer is increasing in people under the age of 50. We found that most colorectal cancer in young people comes from the left side of the colon. We know that older patients with stage IV left‐sided colorectal cancer live longer and respond better to treatment. We show the same is true in young people. We also compared left‐ and right‐sided cancer in stages I, II and III. Left‐sided colon cancer has better survival in all stages except stage II. We think this is because stage II colon cancers on the right are mismatch repair deficient (MMRd), which take longer time to spread.

## 1 INTRODUCTION

While the overall rate of colorectal cancer (CRC) was decreasing over the past few decades, the incidence of early‐onset colorectal cancer (EOCRC), defined as CRC diagnosed under the age of 50, is rising at an alarming rate. Since 1975, there has been a 67% increase in the incidence of CRC in patients between the ages 20–49.^1^ In 2020, approximately 12% of newly diagnosed CRC cases are expected to occur in individuals under the age of 50 (17,930/147,950).^2^ Most strikingly, the fastest rise in incidence was observed in the youngest age group (20–29 years old).^3^ This has prompted the American Cancer Society to recommend lowering the age of screening for people at average risk to 45.^4^

Screening aside, the oncology community has recognized EOCRC as an emerging unmet need. Specific challenges in EOCRC include a lack of understanding of the etiological drivers behind this epidemiologic increase, and unfamiliarity with survivorship issues in young adults^5^, ^6^ and a dearth of data about whether standard treatments apply to this subset.^7^ The precise causes of the increase in incidence in CRC in young patients are not clear.^8^ Observational and case‐control studies^9^ of risk factors associated with CRC have implicated hereditary syndromes in a minority of EOCRC and have not explained the steep rise. Interestingly, the rise in EOCRC is driven by left‐sided tumors.^3^, ^10^, ^11^ Additionally, a site‐specific distinct molecular signature in EOCRC is emerging. Some studies suggested various potential risk factors for EOCRC as diet, stress, gut microbiota, and many others.^12^

Primary tumor sidedness (PTS) is an independent prognostic factor in metastatic CRC.^13^, ^14^, ^15^, ^16^ PTS is also a predictive factor for response to EGFR inhibition in stage IV CRC, and laterality has been incorporated in the current version of National Comprehensive Cancer Network (NCCN) guidelines^17^ as a surrogate for response. These recommendations are based on results from the German‐Austrian FIRE‐3^18^, ^19^ and the US CALGB‐80405^20^ trials which tested FOLFOX/FOLFIRI in combination with bevacizumab versus cetuximab and found that right‐sided colon cancer (RCC) derives less benefit from cetuximab than left‐sided colon cancer (LCC). Embryologically, the right colon is derived from the midgut, while the left colon arises from the hindgut suggesting varied tumor biology. Hence, tumors arising from different embryological states are associated with distinct genetic drivers (RCC: BRAF mutation, MMRd, CpG island methylator phenotype CIMP vs. LCC: chromosomal instability, KRAS mutation, APC mutations), and ultimately different responses to systemic therapies. Nevertheless, data regarding the impact of PTS in the non‐metastatic setting are lacking. To the best of our knowledge, there are no data on PTS in the Veterans Affairs (VA) population. In this study, we aim to investigate the role of PTS in all stages with a focus on EOCRC and in the setting of the VA health‐care system.

## 2 METHODS

Nationwide data from the National Veterans Affairs Cancer Care Cube Registry (CCCR) were analyzed. No patient charts were accessed. The main data source for the CCCR is the Oncology Domain tables on the Corporate Data Warehouse (CDW) raw server, which is updated every 2 weeks. The Oncology Domain tables are created from the VISTA OncoTrax software package. The registry was accessed on 12 August 2017 and 2 June 2018, and data input after this date is not included in this study. Unique cases of CRC with accession year between 2001 and 2015 were analyzed.

ICD codes C18 to C20 were used to delineate patients with RCC vs. LCC. RCC was defined as cancer from the cecum to the hepatic flexure (ICD C18.1–C18.3), while LCC was defined as cancer from the splenic flexure to the rectum (ICD C18.5–18.7 & C19 & C20) with transverse cancer in between flexures (ICD C18.4). ICD codes C18.8 and C18.9 referring to overlapping and unspecified parts of the colon, respectively, were excluded. The registry defines unique cases as those with the same combination of the following data points: patient social security number, diagnosis date, primary tumor site, sequence number, histology ICD 03 Code, grade differentiation ID, and laterality. Accession year refers to the year in which the patient was first seen at the reporting institution for diagnosis and/or treatment of the primary cancer recorded. The registrar further classifies cases based on abstract status. "Complete" abstract status indicates that all data points have been entered by the Tumor Registrar for that particular case. Only cases with complete abstract status were considered for this study. After application of the above qualifiers (unique cases, complete abstract, ICD code, all stages, and accession year span 2001–2015), 65,940 total cases of CRC were identified.

Demographic data on the CCCR including age at diagnosis, gender, and survival were generated from the VA Health Eligibility Center (HEC) demographic file. Survival in the CCCR was defined as <1 year, 1–5 years, 5–10 years, 10–15, and >15 years. Race and ethnicity were derived from the CDW. Demographic characteristics including race and ethnicity were determined based on information provided by patients at initial contact with the VA hospital. Local IRB approval was obtained for the study.

Microsoft Excel (Microsoft Corp) was used for data tabulation and graph formulation. Discreet data points were described using percentages and compared using Chi‐squared test with two‐sided *p*‐value of <0.05 considered as statistically significant. Graph slopes and Chi‐squared tests were calculated using VassarStats.

## 3 RESULTS

### 3.1 Demographic data and age distribution

Of the 65,940 CRC cases diagnosed at the VA between 2001 and 2015, 19,969 (30.28%) cases originated from the RCC, while 38,754 (58.77%) originated from the LCC. Expressed as a left to right (L:R) ratio of 1.94, LCC is twice as common as RCC in the VA population. Transverse colon is rare with only 6.36% (4,191) cases. Only 4.59% of CRC cases (3,026) were excluded as they were documented as originating from overlapping or unspecified parts of the colon. The demographic data of the VA Cancer Care Cube are described in Table 1.

**TABLE 1:** Baseline characteristics of the VA Cancer Cube Colorectal Cancer Population (2001–2015)

	*n*	Right	Left	Transverse	Excluded	L:R ratio
*n* (%)	65,940	30.28% (19,969)	58.77% (38,754)	6.36%(4191)	4.59% (3026)	1.94
Gender						
Male	97.56% (64,334)	30.13% (19,386)	58.93% (37,913)	6.36% (4091)	4.58% (2944)	1.96
Female	2.43% (1602)	36.33% (582)	52.31% (836)	6.24% (100)	5.12% (82)	1.44
Race						
White	68.80% (45,366)	30.16% (13,684)	59.36% (26,928)	6.34% (2874)	4.14% (1880)	1.97
Black or African America	17.03% (11,229)	31.88% (3580)	55.98% (6286)	6.97% (783)	5.17%(580)	1.76
Native Hawaiian or other Pacific islander	0.61% (403)	29.03% (117)	59.06% (238)	6.70% (27)	5.21% (21)	2.03
Multiple races	0.75% (495)	26.06% (129)	63.43% (314)	5.05% (25)	5.45% (27)	2.43
American Indian or Alaskan Native	0.49% (326)	31.60% (103)	59.82% (195)	6.13% (20)	2.45% (8)	1.89
Asian	0.35% (229)	22.71% (52)	66.81% (153)	3.49% (8)	6.99% (16)	2.94
Unknown/declined	11.97% (7892)	29.19% (2304)	58.79% (4640)	5.75% (454)	6.26% (494)	2.01
Ethnicity						
Not hispanic or latino	83.83% (55,275)	30.47% (16,845)	58.73% (32,464)	6.54% (3617)	4.25% (2349)	1.93
Hispanic or latino	5.06% (3335)	28.37% (946)	61.05% (2036)	4.80% (160)	5.79% (193)	2.15
Unknown/declined	11.12% (7330)	29.71% (2178)	58.04% (4254)	5.65% (414)	6.60% (484)	1.95

As expected in a military setting, the VA CRC population is predominantly male (97.56%). Among the 1,602 women veterans, RCC constituted 36.33% of cases compared to only 30.13% in men (*p* < 0.0001). The described population is predominantly White with 68.80% (45,366) identifying as such. The second most common group is Blacks with 17.15% (11,229) of the population. When afflicted with CRC, Asians are almost three times more likely to have LC than RC as opposed to two times overall (Asian L:R 2.94, Overall L:R 1.94, *p* = 0.0113). Conversely, Blacks are more likely to have CRC from the right side of the colon and less from the left side (Blacks L:R 1.76, Overall L:R: 1.94, *p* < 0.0001). Whites, American Indians/Alaskan Natives, and Native Hawaiians/Pacific islanders have similar rates to the general population; as do the unknowns and patients who declined to answer, indicating no self‐selected bias. Overall, only 5.06% of the population identified as Hispanic or Latino. Interestingly, Hispanics are more likely to be afflicted with left‐sided colon cancer with 61.05% of all CRC (L:R ratio of 2.15; *p* = 0.0108).

### 3.2 Age distribution

**FIGURE 1:**
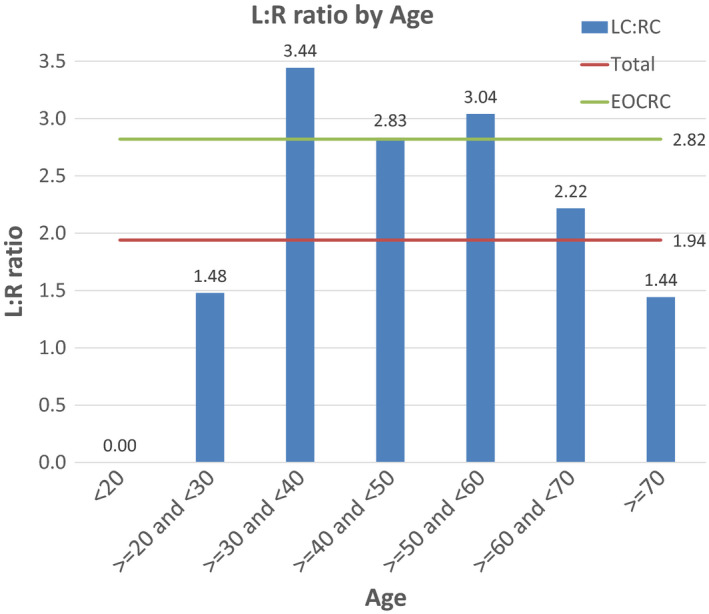
Left to Right ratio (L:R) by age at diagnosis. The average L:R for CRC and EOCRC is represented by continuous lines

Incidence of EOCRC, defined as CRC before the age of 50, constitutes 3.18% of CRC database with most EOCRC occurring in the fourth decade. The laterality distribution in EOCRC is skewed significantly to the left with a L:R ratio of 2.82 (*p* < 0.0001) (Table 2). Table 2 further describes the age distribution for LCC and RCC. RCC affects the elderly disproportionately with just over half of cases (51.78%) occurring in patients over the age of 70. This contrasts with LCC as only 38.46% occur over the age of 70 (>70 L:R of 1.44, overall L:R 1.94, *p* < 0.0001). Figure 1 illustrates the laterality of CRC by age distribution. CRC in every age category is more likely to be left‐sided (L:R > 1) with an average L:R ratio of 1.94, except for the category <20 where both cases are right‐sided. There are not enough cases to draw statistically significant conclusions in the <20 and 20–30 age groups (*p*‐values of 0.2035 and 0.1839, respectively). Starting at the age of 30, patients affected by CRC are more likely to have LCC the younger they are: CRC patients between 30 and 50 are almost 3x more likely to have LCC than RCC (L:R of 3.44, 3.82, and 3.04 for the third, fourth, and fifth decade), while CRC occurring over the age of 60 is almost 2× more likely to be LCC (L:R of 2.22 and 1.44 for PTS 60–70 and >70, respectively). RCC affects the elderly disproportionately with just over half of total right‐sided tumors (51.8%) occurring in patients over the age of 70, compared to 38.5% of LCC.

**TABLE 2:** Age distribution of the VA Colorectal Cancer population

Age	Total	Right	Left	Transverse	Excluded	L:C ratio	*p*‐value
<20	0.00% (2)	0.01% (2)	0.00% (0)	0.00% (0)	0.00% (2)	0.00	N/A
>=20 and <30	0.10% (63)	0.12% (23)	0.09% (34)	0.02% (1)	0.17% (5)	1.48	N/A
>=30 and <40	0.40% (261)	0.26% (52)	0.46%(179)	0.36% (15)	0.50% (15)	3.44	0.0024
>=40 and <50	2.68% (1770)	2.09% (418)	3.06% (1184)	2.15% (90)	2.58% (78)	2.83	<0.0001
>=50 and <60	18.23% (12,023)	13.45% (2685)	21.06% (8163)	14.08% (590)	19.33% (585)	3.04	<0.0001
>=60 and <70	35.04% (23,103)	32.24% (6439)	36.83% (14,273)	34.19% (1433)	31.66% (958)	2.22	<0.0001
>=70	43.50% (28,684)	51.78%(10,339)	38.46% (14,905)	49.18% (2061)	45.57% (1379)	1.44	<0.0001
Total	100.00% (65,940)	30.28% (19,969)	58.77% (38,754)	6.36%(4191)	4.59% (3026)	1.94	
EOCRC	3.18% (2096)	23.62% (495)	66.65% (1397)	5.06% (106)	4.68% (98)	2.82	<0.0001

Thirty‐four cases were logged in as unknown age.

*p*‐values are calculated as relating compared to the total number of colon cancer by location in a 2 × 4 Chi‐square test.

### 3.3 Stage distribution

**FIGURE 2:**
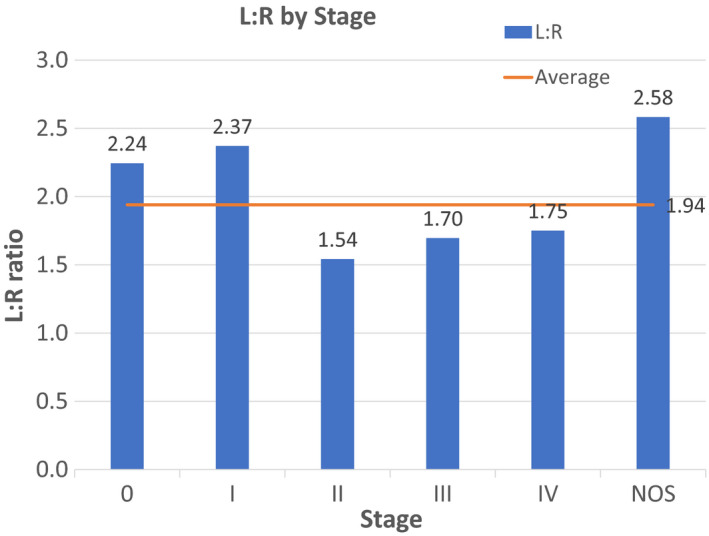
Left to Right ratio (L:R) by performance status. The average L:R for CRC is represented by continuous lines

Stage distribution of CRC at diagnosis is shown in Table 3 and Figure 2. CRC is most likely to be stage I (25.49%) at diagnosis, followed by stages II (19.89%), III (18.26%), and IV (15.94%). Stage 0 (carcinoma in situ) is the least frequent stage with 9.20% of all CRC. LCC is more likely to present in early stages, specifically stage 0 (L:R 2.24) and stage I (L:R 2.37) compared to the average L:R of 1.94. LCC remains more common than RCC at late stages but as the stage progresses so does the proportion of RCC, with L:R ratios of 1.54 at stage II, 1.7 at stage III, and 1.75 at stage IV.

**TABLE 3:** Stage distribution of the VA Colorectal Cancer population

Stage	Total	Right	Left	Transverse	Excluded	L:R ratio	*p*‐value
0	9.20% (6066)	8.31% (1659)	9.61% (3723)	11.83% (496)	6.21% (188)	2.24	<0.0001
I	25.49% (16,808)	22.82% (4556)	27.87% (10,801)	23.79% (997)	15.00% (454)	2.37	<0.0001
II	19.89% (13,117)	23.07% (4606)	18.33% (7102)	24.79% (1039)	12.23% (370)	1.54	<0.0001
III	18.26% (12,042)	20.44% (4081)	17.86% (6922)	17.28% (724)	10.41% (315)	1.70	<0.0001
IV	16.94% (11,167)	17.33% (3461)	15.63% (6058)	13.53% (567)	35.72% (1081)	1.75	<0.0001
NOS	10.22% (6740)	8.04% (1606)	10.70% (4148)	8.78% (368)	20.42% (618)	2.58	<0.0001
Total	65,940	19,969	38,754	4191	3026	1.94	

The Eastern Cooperative Oncology Group Performance Status (ECOG‐PS) had been documented for 26.51% (17,479/65,940) of CRC cases (data not shown). Slightly more than half (54.25%) had an ECOG‐PS of 0, followed by an ECOG‐PS1 of 1 33.84%. LCC has a higher proportion of ECOG‐PS of 0 (55.87%) than RCC (52.89%; *p* 0.001), while RCC had a larger share of worse functional status: ECOG‐PS 2 (RCC: 7.65% vs. LCC: 6.57%; *p* < 0.001); ECOG‐PS 3 (RCC: 4.01% vs. LCC: 3.19%; *p* < 0.001) and ECOG‐PS 4(RCC: 0.95% vs. LCC: 0.61%; *p* < 0.001). For CRC, PTS was found to have a specific functional status: as functional status worsens, so does the likelihood of having RCC.

### 3.4 Overall survival (OS)

**FIGURE 3:**
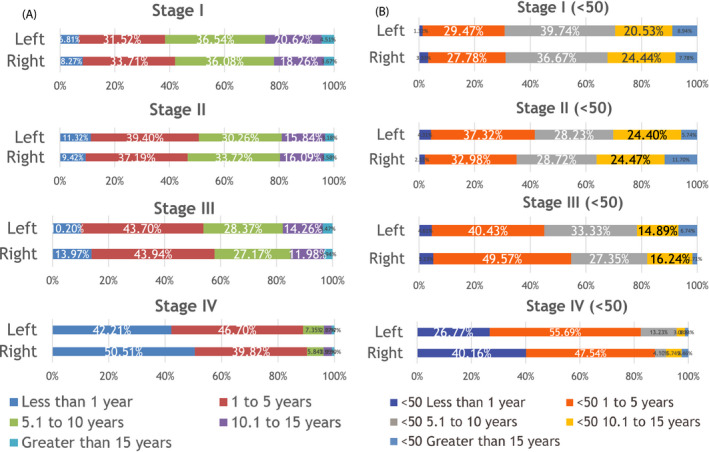
Survival of LCC and RCC by stage in the overall population (A) and patients under the age of 50 (B)

In EOCRC (Table 4B), RCC carries a significantly worse survival than LCC in the metastatic setting (1‐year OS‐RCC: 59.84% vs. LCC: 73.23%; *p* = 0.0086). In the non‐metastatic setting, the difference in 5‐year Overall Survival observed with CRC is not statistically significant in any stage. More detailed visual representations of overall survival of CRC and EOCRC are presented in Figure 3A and B, respectively. In EOCRC, there is no statistically significant difference between LCC and RCC in stages 0–III. Stage IV LCC in patients <50 has a 1‐year OS of 73.23% (vs. 59.84% for RCC).

**TABLE 4:** Overall survival of CRC by sidedness in the overall VA population and in patients under the age of 50

Stage	OS	RCC	LCC	*p*‐value
0	5‐year OS	58.11% (964)	64.33% (2395)	<0.0001
I	5‐year OS	58.01% (2643)	61.67% (6661)	<0.0001
II	5‐year OS	53.39% (2459)	49.28% (3500)	<0.0001
III	5‐year OS	42.10% (1718)	46.10% (3191)	<0.0001
IV	1‐year OS	49.49% (1713)	57.79% (3501)	<0.0001
Total	—	30.28% (19,969)	58.77% (38,754)	—

Stage	OS	RCC<50	LCC<50	*p*‐value
0	5‐year OS	80.00% (16)	64.60% (73)	0.2753
I	5‐year OS	68.89% (59)	69.21% (209)	0.6033
II	5‐year OS	64.89% (61)	58.37% (122)	0.3428
III	5‐year OS	45.30% (53)	54.96% (155)	0.0991
IV	1‐year OS	59.84% (73)	73.23% (238)	0.0086
Total		23.62% (495)	66.65% (1397)	—

In CRC (Table 4A), metastatic tumors originating from the right are associated with significantly worse 1‐year overall survival (RCC: 49.49%) than those originating from the left (LCC: 57.79%) (*p* < 0.0001). Similarly, LCC has a better 5‐year overall survival than RCC for stage 0 (RCC: 58.11% vs. LCC: 64.33%; *p* < 0.0001), stage I (RCC: 68.01% vs. LCC 61.67; *p* < 0.0001), and stage III (RCC: 42.10% vs. 46.10%; *p* < 0.0001). Surprisingly, stage II right‐sided colon tumors carried a higher 5‐year OS than the left‐sided ones (RCC: 53.39% vs. LCC 39.28%; *p* < 0.0001).

## 4 DISCUSSION

### 4.1 Epidemiology

This paper describes the first study of PTS in Veterans Affairs hospitals. Despite the overwhelming White male slant of the Veterans population, it reinforces ^21^, ^22^, ^23^ that RCC is more likely to arise in women, Blacks, and the elderly. We also demonstrate that RCC presents at a more advanced stage, often with worse performance status than LCC. Despite the increase in diagnosis of CRC on routine screening colonoscopies, most CRC still presents symptomatically.^24^ Symptoms of CRC differ by tumor location. Symptoms more characteristic of RCC are anemia and vague abdominal pain, while LCC usually presents with hematochezia, change in bowel habits, and is more likely to cause obstruction. This difference is thought to be due in part to the larger luminal diameter of the cecum and consistency of the bowel contents, as the tumors need to grow large enough to cause obstructive symptoms. Traditionally, this anatomical discrepancy was thought to explain the shortened survival associated with RCC. The predominance of RCC in specific epidemiologic subpopulations (Blacks, women, and elderly) belies that explanation.

### 4.2 Molecular genetics

More recently, molecular studies revealed that the pathophysiology of the malignancy and not just the anatomy of the digestive system carries prognostic and predictive implications. Tumors in each colonic segments (RC and LC) with distinct embryological origins (midgut and hindgut) express different molecular genetics^25^, ^26^ and ultimately exhibit varied responses to systemic therapies. Right‐sided tumors are more likely to express BRAF mutations, have MMRd, and CpG island methylator phenotype (CIMP) mutations, while LCC is more associated with K‐RAS mutations, chromosomal instability, and defective tumor suppressors (p53, adenomatous polyposis coli [APC], and Deleted in Colon Cancer [DCC]).^27^, ^28^ A consortium analysis^29^ of CRC revealed that the consensus molecular subtypes (CMS) type 1 (CMS1) tumors were more frequently right‐sided, while CMS2 tumors were more likely to be left‐sided.

### 4.3 Overall survival

We show metastatic LCC has significantly better survival than RCC, as previously demonstrated in the CALGB80405^30^ and FIRE‐3^19^ studies. We did not evaluate the impact of EGFR‐ and VEGF‐directed therapies in our analysis. The impact of PTS in the non‐metastatic setting is less well‐studied. To our knowledge, this is the largest retrospective review of PTS not performed within the confines of the SEER database and the third largest overall. Given that the treatment of early stage disease is primarily surgical, the difference in survival is expected to be less pronounced. Nonetheless, LCC is associated with better OS in all stages except stage II, where, interestingly, RCC performed slightly better. Previous studies using the SEER database yielded mixed results for RCC vs. LCC in stage II. Studies by Yang^31^ (2000–2012) and Weiss^32^ (1992–2003) found a survival benefit favoring RCC in stage II, while Warschkow^33^ (2004–2012) and Meguid^34^ (1988–2003) found LCC to have a longer survival. Prior analyses of molecular markers by stage and side have shown that right‐sided stage II adenocarcinoma is enriched in MSI‐high^35^ and MMRd^36^ tumors. This difference might help explain the more favorable prognosis of stage II RCC. The differential that PTS confers in survival has implications for adjuvant therapy in locoregional disease. Current factors^37^ used to define high‐risk stage II CRC eligible for adjuvant chemotherapy include T4 tumors, obstruction, perforation, lymphovascular invasion, undifferentiated histology, and the retrieval of less than 12 lymph nodes during surgery. Whether laterality in stage II imparts benefit from adjuvant treatment has not been investigated in a prospective fashion. The seminal IDEA trial^38^, ^39^ has established the duration of adjuvant therapy in stage III CRC. High‐risk groups benefitting from longer duration of FOLFOX, but not CAPOX, were defined as T4 or N2, regardless of PTS. The role PTS plays in determining length of adjuvant treatment in stage III has not been studied. More recently, trials sought to incorporate liquid biopsies and next‐generation sequencing into the management decisions of stage II and III CRC. The established role of PTS as a surrogate for response in the metastatic setting may herald a shift to the left in the local setting. This is likely to be especially relevant in developing countries and rural practices where scarce resources would not allow for the routine use of precision oncology.

### 4.4 EOCRC

We report that EOCRC accounts for 3.18% of all cases of CRC in the VA population. An analysis of the National Cancer Data Base (NCDB) during the same timeframe (2004–2015) reports 11% of CRC to be diagnosed before the age of 50.^40^ The lower fraction of EOCRC observed in our study can be accounted for by the higher incidence of malignancies in veterans than the general population.^41^ On average, veterans are more likely to be older, smoke, drink alcohol, and to have been exposed to Agent Orange.

While CRC is almost twice as likely to originate from the left colon in the overall population, EOCRC is almost three times as likely to arise from the left side (L:R ratio 2.84). This ratio is highest for cases diagnosed in patients in their 30 s (L:R of 3.44). The predilection of CRC to arise from the distal colon is consistent with prior observations.^42^, ^43^ One potential explanation of this tendency is the notable difference in transit time in each colonic segment by age.^44^, ^45^ A relatively longer rectosigmoid transit in adolescents can lead to prolonged exposure to potential carcinogens, such as high‐fat food and processed meat.^46^, ^47^

A consensus^48^ is emerging that EOCRC is a pathologically, epidemiologically, anatomically, and biologically different disease than late‐onset CRC. We sought to examine the impact of laterality in EOCRC in this study. In stage IV, LCC exhibits a longer 1‐year OS than RCC in younger patients, similar to the overall population. While the difference in 5‐year overall survival—favoring LCC in stages 0, I, and III and RCC in stage II—is not statistically significant, there remains a trend toward better 5‐year OS in stage II RCC and stage III in EOCRC. The smaller number of patients with EOCRC could account for this difference. Due to their young age, EOCRC patients are more likely to receive more intensive chemotherapy than their elder counterparts.^49^ Unfortunately, this practice has not resulted in a commensurate increase in overall survival. Identification of specific prognostic and predictive markers is of paramount importance to guide therapy. Recent molecular analyses by sides in EOCRC^12^, ^50^ have sought to shed light on these questions.

Our study is limited by the inherent biases of its retrospective design. We acknowledge the lack of availability of data regarding molecular profiling, follow‐up, and treatment modalities as limitations. The data presented here may show a need for a greater incorporation of PTS in the design of prospective randomized clinical trials. The rise in EOCRC dictates a greater involvement of these affected in clinical trials.

## CONFLICT OF INTEREST

Anthony Frank Shields: Consulting: Caris Life Sciences, Lexicon. Speaker's Bureau: Caris Life Sciences. Travel, accommodations, expenses: Caris Life Sciences, Lexicon, Nouscom. Research funding: Alkermes, Astellas, Astra Zeneca, Bayer, Boehringer Ingelheim, Boston Biomedical, Caris Life Sciences, Daiichi, Eisai, Esperas Pharma, Exelixis, Five Prime Therapeutics, H3 Biomedicine, Halozyme, Inc, ImaginAb, Inovio, Incyte, Jiangsu Alphamed, Lexicon, LSK BioPartners, Inc, MSK, Nouscom, Plexxikon. Shanghai HaiHe, Taiho, Torque, Xencor.

Philip Agop Philip: Consulting: Celgene, Ipsen, Merck, TriSalus Life Sciences, Daiichi Sankyo, SynCore, Taiho Pharmaceutical. Speaker's Bureau: Celgene, Bayer, Ipsen, Novartis, Incyte. Travel, accommodations, expenses: Rafael Pharmaceuticals, Celgene, AbbVie. Honoraria: Celgene, Bayer, Ipsen, Merck, AstraZeneca, TriSalus Life Sciences, Blueprint Medicines, SynCore, Array BioPharma. Research funding: Bayer, Incyte, Karyopharm Therapeutics, Merck, Taiho Pharmaceutical, Momenta Pharmaceuticals, Novartis, Plexxikon, Immunomedics, Regeneron, Genentech, Tyme, Caris Life Sciences, ASLAN Pharmaceutical, QED Therapeutics, Halozyme, Boston Biomedical, Advanced Accelerator Applications, Lilly, Taiho Pharmaceutical**,** Merus. Uncompensated relationships: Rafael Pharmaceuticals, Caris MPI.

## Data Availability

In accord with VA rules and regulations and as stated in our IRB, data will not be available for sharing.
